# Spotlight on Biomimetic Systems Based on Lyotropic Liquid Crystal

**DOI:** 10.3390/molecules22030419

**Published:** 2017-03-07

**Authors:** Juliana F. de Souza, Katiusca da S. Pontes, Thais F. R. Alves, Venâncio A. Amaral, Márcia de A. Rebelo, Moema A. Hausen, Marco V. Chaud

**Affiliations:** 1 Laboratory of Biomaterials and Nanotechnology, University of Sorocaba (UNISO), Sorocaba, SP 18078-005, Brazil; julianafsz@yahoo.com.br (J.F.d.S.); katiusca.pontes@outlook.com (K.d.S.P.); thaisfrancine1@hotmail.com (T.F.R.A.); venancio_mt@hotmail.com (V.A.A.); marciarebelo@ig.com.br (M.d.A.R.); 2 Laboratory of Post-Graduate Program in Biotechnology and Environmental Monitoring (PPGBMA), University of São Carlos (UFSCAR), Sorocaba, SP 18052-780, Brazil; mahausen@pucsp.br; 3 Laboratory of Biomaterials (LABIOMAT), Pontificial University Catholic (PUC), Sorocaba, SP 18030-070, Brazil

**Keywords:** liquid crystals, lyotropic liquid crystals, biomimetic systems, immunodetection, drug carriers

## Abstract

The behavior of lyotropic biomimetic systems in drug delivery was reviewed. These behaviors are influenced by drug properties, the initial water content, type of lyotropic liquid crystals (LLC), swell ability, drug loading rate, the presence of ions with higher or less kosmotropic or chaotropic force, and the electrostatic interaction between the drug and the lipid bilayers. The in vivo interaction between LCC—drugs, and the impact on the bioavailability of drugs, was reviewed. The LLC with a different architecture can be formed by the self-assembly of lipids in aqueous medium, and can be tuned by the structures and physical properties of the emulsion. These LLC lamellar phase, cubic phase, and hexagonal phase, possess fascinating viscoelastic properties, which make them useful as a dispersion technology, and a highly ordered, thermodynamically stable internal nanostructure, thereby offering the potential as a sustained drug release matrix for drug delivery. In addition, the biodegradable and biocompatible nature of lipids demonstrates a minimum toxicity and thus, they are used for various routes of administration. This review is not intended to provide a comprehensive overview, but focuses on the advantages over non modified conventional materials and LLC biomimetic properties.

## 1. Introduction

Rationally designed materials that explore specific, adjustable, reversible, and biodegradable interactions offer advantages over non modified conventional materials, for both regenerative medicine and controlled or target-specific drug delivery systems. When designed to detect and respond to physiological stimuli or imitate structural and functional aspects of biological signaling, these materials are denominated as biomimetic [1]. Biomimetic, biomimicry, or bionic materials are designed to imitate the native temporal and spatial presentation of biochemical and physiological factors, or replace them, using a “like with like” approach in order to improve or restore biological functions. Biomimetic material design only requires the modeling of one or more functional principles, from any biological system. One approach to biomimetic concept creation is to first summarize the essential function of the desired system and then obtain a biological system that serves as a model and performs similar functions [2].

Liquid crystalline phase structures are often found in living animals, plants, and bacteria. Cellular membranes, for example, are the result of lyotropic liquid crystalline phase, resulting from the dissolution of phospholipids in water. Membrane curvature plays a crucial role in its performance, especially in cell signaling and traffic, and the release of mycobacterial lipids [3,4]. In 1991, Chazotte et al. showed that ubiquinone (also known as Coenzyme Q) moves laterally and transversely in a liquid crystalline membrane, but mostly remains in the membrane's acyl chain region [5]. In addition, evidence indicates that the mitochondrial microsomal membrane and tight junctions between cells are formed by a non-lamellar, liquid crystalline structure. Detailed studies on the properties of methylated or non-methylated sterile esters have shown that a large number of these compounds are intermediates in the biosynthetic sequence, leading to the formation of sterols (sitosterol and stigmasterol), whose properties have been related to crystalline liquid properties that are essential for some plant biochemical processes (since sterile ester can form mesophases) [6].

The stress responses to extreme temperatures are essential for the survival of several organisms, and it has been established that in low temperatures, lipid microviscosity is crucial for a microorganism’s survival [7]. When analyzing the protein structural alteration rate, hydrogen-deuterium exchange, membrane-lipid disorder, and membrane–water interfacial order/hydration as functions of temperature, Laczkó-Dobos and Szalontai concluded that the gel-to-liquid lipid crystalline phase transition is related to the temperature of cell growth in wild types, but not in desA-/desD mutants [7].

Different studies have shown the existence of many liquid crystalline structures, with lamellar organization being only one. When a phospholipid extracted from a human brain was analyzed using the same technique, two liquid crystalline phases were found: one formed by lipid and water layers in an ordered sequence (lamellar phase) and the other formed by a hexagonal arrangement of cell cylinders. Finally, each cylinder is a thin water channel covered by the hydrophilic groups of lipid molecules (hexagonal phase), with hydrocarbon chains filling the gap between them, and both coexist in equilibrium [8,9,10].

Alejandro D. Rey published a review on the phase transition, self-organization, interfaces, defects, rheology, and integrated application of biological mesophases and biological liquid crystal (BLC) models, clearly indicating rationally structured molecules in the form of liquid crystals (LC). Imitated organic LC types are analogous, wherein the orientational sequence is frozen in solid form (mammalian connective tissues, fish dermal scales, insect and crustacean exoskeletons, and plant cell walls); biopolymer solution (collagen, cellulose, chitin, viral suspension, and *Salmonela typhimurium* flagella); and in vivo mesophases (DNA, bacterial chromosome, egg shell collagen, spider silk, sickle cell hemoglobin, and synovial fluid). This classification, although ambiguous, is used to differentiate the origin of the BLC [11,12,13,14]. Integrated modeling applications involving thermodynamics/defects/rheology for the experimental characterization of different BLC, including DNA and collagen solutions, have been shown to provide conceptual organization and quantitative tools to establish the properties of these natural occurring materials [15]. Although lamellar liquid crystalline phase (Lα) is the natural state of cell membranes, it can be divided into two distinct subclasses: ordered (lo) or disordered (ld) liquid states [16]. Ordered and disordered, in these terms, refer to a lipid’s acyl chain of state. The term “liquid” herein refers to the lack of long-range positional order, and consequently, to a “lack of the lattice” within the bilayer plane and to high-speed lateral diffusion; in contrast to gel phase, such as Lβ, which, in this terminology, is considered to be “solid,” with a positional order within the membrane and very slow lateral diffusion [17,18].

## 2. Biological Liquid Crystals

BLC exhibit long-range partial positional and orientational sequences, and, similar to their synthetic counterparts, are structural and functional materials that display crystal anisotropic elastic behavior and anisotropic suspension viscous behavior [19]. The BLC are found, for example, in nucleic acids, proteins, carbohydrates, and fats [20], and, as synthetic LC, are sensitive to electromagnetic fields, pH, substrate and interfaces, temperature, light intensity, ultrasound, and concentration gradients [21,22,23,24,25]. These studies demonstrate the potential of molecular design to create new functional materials with predictable operating modes, owing to a LC sensitivity to endogenous and exogenous stimuli, which enables drug release in response to different triggers [26]. Among the exogenous triggers, light intensity is advantageous, since it can be applied remotely with spatial and temporal precision, ensuring high levels of drug control and toxicological safety. Lipidic mesophases (LMP) are systems for alternative drug release that can be triggered by external stimuli [27]. Aleandri et al. recently described light-responsive LLC synthesis using an LMP system comprising monoolein, oleic acid, and azobenzene-derived lipids as the photoactive unit [28].

## 3. Lyotropic Liquid Crystals-Based Emulsion

Emulsions are dispersing and thermodynamically unstable systems, comprising mutually immiscible liquids and a surfactant. The physical properties of emulsified systems depend on the correct surfactant choice and concentration, the dispersed droplet size, the dispersed and continuous phase’s concentration, and its preparation technique. In addition to the influence on the physical stability, the dispersed droplet size also influences the active conveyed emulsions’ bioavailability. The droplet size can be altered by dispersed phase slight coalescence and changes in the bioavailability of drugs and nutraceuticals, depending on the emulsion preparation time [29,30].

Because of their industrial, scientific, and drug-release-related importance, many research efforts have been conducted to maintain emulsion-free, energy-high potential, while maintaining their physical and chemical stability, preventing or minimizing coalescence, flocculation, or sedimentation. Different studies have shown that the physical and structural properties of emulsion can be adjusted by using different types of continuous phases, such as the gelled, continuous phase [31], continuous crystalline phase [32,33], particles [34], thermotropic liquid crystal [35,36], and LLC [37,38], or self-emulsified delivery systems (SEDDS) [39,40].

Among the different types of continuous emulsion-phase, LLC are the most versatile structures, enabling the production of mesoporous or macroporous material by a simple, single-step method, through the modeling of highly concentrated emulsions with liquid cubic crystals in the continuous phase [41,42]. Alam and Aramaki published a review on the progress and prospects of LLC, based on emulsions. In the present article, the authors summarize the recent development in oil/liquid crystal or water/LC emulsions’ formation, stability, and rheology [43].

The type of molecular structure that generates a liquid crystalline phase is amphiphilic. Amphiphilicity involves the coexistence of two completely different molecule realities, in relation to water. On one hand, flexible hydrocarbon chains prevent contact with the water, while a polar head group tends to orient toward water [44,45]. An amphiphilic self-organized system aggregates through a self-organization process, directed by the hydrophobic effect when mixed with water. Amphiphilic self-organized systems are characterized by structures in which the polar part (hydrophilic group) protects the nonpolar chain (hydrophobic group) from contact with the aqueous solvent [46]. When the amphiphilic concentration in a lyotropic system exceeds the micelle critical concentration or the critical aggregation concentration (CAC), an amphiphilic self-organized aggregate is formed as an independent entity, in balance with an amphiphilic monomer in the solution [47], without any orientational or positional long-range order [44].

Liquid crystalline phases with different architectures are formed when the amphiphilic concentration in the water exceeds CAC, and this compound self-organizes in a spatially regular manner. The self-organized system depends on a delicate balance between intermolecular forces to promote the structure of LC, and even a small alteration can disrupt the ordered structures of LC [48]. Amphiphilic self-organization in aqueous or non-aqueous medium also depends on the temperature and the degree of hydrophobic chain introduction [49,50,51]. LC have different phases known as mesophases, which differ in the individual orientation of their molecules, with different orders in each phase. The most commonly described phases are the crystalline lamellar or amorphous, hexagonal types H_1_ and H_2_, and discontinuous (I_1_ and I_2_) and bicontinuous cubic types (V_1_ and V_2_) [22,52,53]. Each stage is sensitive to temperature, pH, and the presence of impurities or foreign particles. Therefore, LC can be considered responsive materials [54]. Regarding LC orientational changes, not only the nature of foreign particles, but also their shape, size, and surface properties, play a critical role in the manufacturing process. Thus, LC materials that have optical anisotropy when observed under polarized light may have a dark or bright appearance, according to their order or orientation [48].

Molecular orientation in LC is the origin of the birefringence that is not present in the glassy state, as it does not have the molecular orientational order. LC show general properties like birefringence; a response to magnetic and electric fields; optical activity in twisted nematic phases; and sensitivity to temperature, resulting in color changes [55]. An aligned LC phase shows two refractive indices, along and across the long molecular axis. When a sample is illuminated, it is split into two components: ordinary (polarized perpendicular) and extraordinary (polarized parallel) rays. The difference in the refractive index between the two directions is the birefringence. The extraordinary refractive index (n_e_) is bigger than the ordinary refractive index (n_o_). Nearly all LC have a positive birefringence. The birefringence decreases with temperature, due to a reduction in the order parameter with increasing temperature. The birefringence’s optical property can be defined as the resolution of a light wave into two unequally reflected or transmitted waves, by an optically anisotropic medium [56].

The dielectric constant of an aligned LC phase depends on the direction of the molecules within the sample. In an electric field, the higher dielectric constant will tend to assume a horizontal position on the supporting surface. The dielectric property of the liquid crystalline system can be defined as the material that does not readily conduct electricity [55,56].

## 4. Viscoelastic Properties

Only a small number of materials obey Newton’s law of viscosity or Hooke’s law of elasticity, and neither are applicable to LC. The complex behavior of rheological materials, which often have both fluid and solid properties, is called viscoelasticity. Yu et al. shows that a typical creep curve consists of three regions: the elastic (first part), viscoelastic (second part), and viscous region (third part), respectively. An instantaneous elastic response, retarded elastic response, and a linear relationship between strain and time, begins at the viscoelastic region to infinity [57]. Further manifestations of viscoelasticity were reported for a lecithin/water binary system after detecting normal stress differences under shear flow, measuring the stress relaxation after steady flow and attempting to conduct small amplitude oscillatory shear experiments [58]. In 1993, Robles Vazquez et al. presented the whole linear viscoelastic spectrum and interpreted it on the basis of a weak-gel response, which implies a preponderance of the elastic component on the viscous one, within the standard available experimental conditions [59]. The conclusion that emerges from this work is that the interaction between electrical double layers of the surfactant molecules in the lamellar bilayers, as well as hydration and van der Waals forces, play an important role in the viscoelastic behaviour of surfactant-based lyotropic mesophases. In 2005, Mezzenga et al. showed that each distinct LC phase has a specific rheological signature. The lamellar phase was demonstrated to be a plastic material, the hexagonal phase to be a viscoelastic material, and the bicontinuous cubic phase showed complex rheological behaviour, with different characteristic relaxation times. They argued that current models describing the viscoelasticity of bicontinuous cubic phases are inadequate for fully explaining the experimental features observed [60].

## 5. Medical Setting

Zhou et al. (2014) and Fong et al. (2016) concluded that LLC materials will continue to evolve as unique systems with advantageous physical, chemical, and biological properties, which will be exploited in a wide range of advanced biomedical material and devices, and which will bring about changes in medical settings [61,62]. Liquid-crystal materials have often been referred to as a curious phases of matter, but their impact on modern technology has been profound [63].

A large subset of the LLC research community is making use of our new understanding of these materials, to develop innovative biosensor devices for bedside diagnostics and laboratory applications. LLC materials, with their birefringent properties and extreme sensitivity to surface interactions, are poised to play an important role in these devices. Optical sensors using LC materials could eliminate the need for markers or tags, as the LLC molecules act to enhance the optical appearance of signals of a biological process or structure. LLC have been recently used in a wide array of sensing applications that exploit the high sensitivity of their alignment to the conditions of surrounding immiscible media [63,64]. Combined with the optical anisotropy of LLC, this sensitivity produces a rapid, easily-visualized response. The potential of LLC as a sensor is significant and the design of the sensor permits a response to be viewed in a small area [64].

Yamaguchi et al., using small angle x-ray scattering (SAXS) analysis, demonstrated that a response to the transient structural change of a lipid was detectedm which might result from the diffusion of oil and/or water from nanocube^TM^ LC, towards the lipid lamella phase. Simultaneously, a significant increase in growth factors and inflammatory cytokines was detected after the administration of nanocube^TM^. Not only the excess expression of cytokines, but also the extent of transepidermal water loss as a barrier marker of skin, increased. These observations suggest that a structural change in a lipid can stimulate and trigger the recognition of a slight injury in the wound defence and a repair response as homeostasis [65]. In addition to traditional polymer processing, biopolymers were fabricated with nanometer scale network morphology, using LLC templates to impart lamellar nanoscale architecture within the synthetic scaffolds. For three-dimensional directional cell growth, hydrogels with oriented channels have been used with some success. However, the microscopic pores usually have no nanoscale orientation for guiding cell growth, and the scaffolds are most often preformed, thus requiring invasive implantation surgery. The aligned nanofibers can direct cell growth. The alignment of LC at low shear rates has also been used to create scaffolds with a nanoscale orientation. Three-dimensional, cell-containing scaffolds with a long-range directional order can be produced by a variety of external force fields (i.e., magnetic field, flow field, and meniscus force). LLC of nanoscale phage particles can be mixed with cells and injected into a matrix, to yield microscale fibers displaying a long-range (greater than 1 cm) ordering of aligned phage. So, constructing tissue regenerating materials with phage nanofibers may provide several advantages over conventional methods [66,67,68]. Responsive, lipid-based mesophase systems offer other opportunities in biomedical applications, such as biosensing, diagnostics, drug delivery systems [62], tissue targeting [69], and tissue regeneration [65,67,70]. Fong et al. (2016) completed the important review on responsive, self-assembled nanostructured lipid systems for drug delivery and diagnostics. They summarized the distinctive features of this LLC and concluded that the promising applications of LLC depend of the synergistic exploitation of the mesophases and the advancement of science [62].

## 6. Liquid Crystals as Sensing Systems

The LC biosensor is a research area of sensor technology, which integrates modern biotechnology, and in recent years, the LC biological sensors have become a hotspot of associated research [71]. A macromolecular organic compound is ordinarily immobilized in close proximity to a transducer surface, facilitating direct or mediated signal transfer to the transducer [72,73]. Different types of biologically sensitive materials can be selectively used to recognize a particular substance. These include enzymes, multi-enzymatic systems, organelles, photosensitive plant and bacterial membranes, protoplasts, whole cells, tissue slices, antibodies, DNA, carrier protein, and even receptor systems isolated from cell membranes [74,75,76]. An acetylcholinesterase biosensor LC system was developed, based on the enzymatic growth of gold nanoparticles for amplified acetylcholine and acetylcholinesterase inhibitor detection [77]. Glucose is an example of an important biomolecule, and the sensor’s ability to detect it and measure its concentration, is important for a better understanding of its role in biological environments such as single cells and bacterial cultures. A glucose sensor based on 4-cyano-4′-pentyl biphenyl and on octadeyltrichlorosilane-coated glass slide as a substrate, showed the sensitivity to detect and measure glucose concentrations from 1.0 pmol to 50.0 nmol [78]. A sensor may be obtained from a biological macromolecular compound, ordinarily immobilized in close proximity to the transducer surface, thereby facilitating signal expansion and direct or mediated transfer to the transducer [48].

Biomimetic devices such as LC can act as signal amplifiers to modulate specific associations, dependent on hydrophobic, electrostatic, and van der Waals interactions. This specificity can be used to detect antigen-antibody, receptor-toxin, enzyme-substrate, and drug-receptor interactions, thereby amplifying the measure of intrinsic activity, transforming the interaction into measurable signals. Valloran et al. (2016) showed that, during the in meso peroxidase enzymatic reaction, the converted product crystallizes within the mesophase domains, generating a detectable birefringence signal. A new general assay principle is presented for the detection of an unprecedented vast class of analytes, using birefringence as a sole optical output signal [79].

Khan and Park showed the use of 4-cyano-4′-pentyl biphenyl as a sensing application material, and demonstrated that the LC-based glucose biosensor functionalized with poly (acrylicacid-b-4-cynobiphenyl-4′-oxyundecylacrylate), and that quaternized poly(4-vinylpyridine-b-4-cynobiphenyl-4-oxyundecylacrylate) has advantages such as low production costs, simple enzyme immobilization, high enzyme sensitivity and stability, and easy polarized optical microscope detection, and may be useful for prescreening the glucose level in the human body [80].

The immunodetection of cancer biomarkers is an essential procedure in cancer detection and diagnosis. The LC-based immunodetection offers an alternative and sensitive approach for the detection of biomarker proteins, with the potential of replacing conventional immunoassays used in biochemical studies. When considering the detection of biomolecules, a higher sensitivity can be achieved through a more significant phase retardation, using a LC mixture of larger birefringence material. Studies on the label-free immunodetection of the cancer biomarker CA125, using larger birefringence material, show that techniques based on birefringent LC have the potential of detecting a wide range of biomolecules and should be further pursued, in addition to conventional immunoassays [81].

The substrate feature for LC alignment is a key component for a LC-based detection system (sensing-LC system). However, the alignment is also influenced by the nature and type of the surfactant molecule at the air-liquid interface. Nonpolar surfactant portion branching and organization influence the liquid crystals’ orientational order (or anchorage). Similarly, ordering/anchoring is dependent on liquid crystals’ alkyl chains and core [82,83]. Studies of the biosensors should focus on adopting new technology and using new materials, selecting the suitable function materials for the determination of the objects. Besides, the bionic biosensors have the potential to replace biological visual, olfactory, gustatory, auditory, tactile, and other sensory organs [71].

## 7. Liquid Crystals Homeotropic Alignment

There are many different types of LC phases, which can be distinguished by their different optical properties. When viewed under a polarized light source, different LC phases appear to have distinct textures. The contrasting areas in the textures correspond to domains where the LC molecules are oriented in different directions. Early on in LC research, observations were made concerning the spontaneous interaction of the fluid with surfaces. Chatelain (1943), in his classic investigation of homogeneous alignment on rubbed surfaces, hypothesized that the orientation results from dipole interactions between an ordered layer of adsorbed fatty impurities and the proximate nematic molecules, but he was unable to eliminate the possibility that the mechanism involved the alteration of the substrate surface itself [84]. Berreman (1972) showed that elastic strain energy may account for the tendency of nematic LC to lie parallel to the direction of rubbing on a solid surface that has been slightly deformed, or perpendicular to a surface that is slightly rough in two dimensions [85]. Homeotropic alignment is the state in which a rod-shaped liquid crystalline molecule is perpendicularly aligned to the substrate, in contrast to homogeneous alignment, in which molecules and substrates are aligned in parallel. This phenomenon of the orientation of liquid LC by surfaces is the so-called anchoring. The phenomenon of anchoring is also similar to the epitaxia observed in the growth of some solid crystals on others. While an epitaxial layer is seldom obtained, LC phases are oriented by any surface, even free surfaces [86].

LLC differ from thermotropic LC in that they are not homogeneous phases. They are obtained by putting rod-like particles or macromolecules in solution, by making soap solutions, or by mixing oil and water by mean surfactants. Amphiphilic surfactant molecules form micelles, with their hydrophilic heads pointing into the water. The hydrophobic tails point into the oil, if it is stabilizing the oil/water interface. All materials showing LC behavior belong to two general classes: lyotropic materials, in which fluid anisotropy results from interactions between anisotropic aggregates of amphiphilic molecules; and thermotropic materials, in which the orientational order arises from interactions among partially rigid anisotropic molecules. Amphiphilic compounds can form a variety of thermotropic LC phases as a function of temperature by themselves, and LLC phases upon the addition of some solvent, such as water or diethylene glycol [87].

The interaction between the surfactant aliphatic alkyl chain and the LC is largely responsible for controlling the orientational arrangement of LC in the LC-water interface [88,89]. The orientational arrangement of LC is also sensitive to the degree of branching and conjugations in aliphatic chains. However, amphiphilic molecules for the LC-water interface are essential for homeotropically aligning the LC [48]. When a synthetic phospholipid is used to align LC molecules, unilamellar lipid vesicles or doped unilamellar lipid vesicles by a receiver, complementary to the target analyte, can also be used to homeotropically align LC molecules [82,90].

The lipid “A” bacterial endotoxin has also been used for LC alignment purposes [68,69]. Sodium dodecyl sulfate is a surfactant used for LC alignment at the air-liquid interface, whose operation is similar to that of phospholipids, but with the advantage that it is used in its unaltered form, where lipid vesicle formation is an essential step to the induction of LC alignment [89]. Ionic surfactants have been used to study the interaction of LC droplets, coated with polyelectrolytes. Bromide octa-decyl-trimethyl ammonium adsorption at the LC-water interface has been undertaken to develop homeotropically aligned LC molecule and immobilized LC droplet optical sensors on living cells [91,92,93]. LC-based sensors have been developed to analyze label-free polyelectrolytes, ions, molecules, and biological systems. Copolymer amphiphilic blocks have been used as sensors [94] for glucose [80] and protein [95]. A protein-containing solution such as bovine serum albumin causes an orientational ordering change of LC sensing dots; this change is indicated by a dark-to-bright transition in LC sensing dots’ optical appearance [96]. The principle of detection of these sensors is based on the LC highly sensitive orientational response to small changes in the surface structure [97].

## 8. Drug Delivery

With the advent of drug design, different lipid types have been studied for drug delivery through different administration routes. Partially hydrophilic lipids in solid state have been used as hydrophilic drug carriers in sustained release systems, in different administration routes, as drug carriers in solid implants, or in biomimetic engineering applications [98,99]. Polar amphiphilic lipid reorganization in water invariably forms lipid bilayers. However, depending on the temperature and on the media’s water content, lipids are organized in cubic lamellar (V_2_) and hexagonal (H_2_) mesophases, which constitute the most common LLC systems used in drug delivery systems (DDS) [91,96]. Lamellar phases (Lα) are formed by higher amphiphilic lipid concentrations and meet a long-range order, whose structure is constituted by a linear arrangement of alternating lipid bilayers and a water channel.

Owing to its potential for controlled drug release, and biomimetic and biopharmaceutical properties, the use of partially hydrophilic lipids in LLC preparation expanded their use in the drug delivery system [14]. At present, LLC are among the most promising strategies for increasing a drug’s bioavailability, and for modifying release and absorption kinetics [28,100,101,102], as well as for target-specific drug delivery systems [69,103]. LLC particles are nanostructures proven to be extremely versatile for DDS, are nontoxic and biodegradable, and can be used for various routes of administration including ophthalmic [104], nasal [105], vaginal [106], dermal [107], oral [108], or parenteral [101].

The structures of the cubic and hexagonal phases have received considerable attention because of their potential as vaccine delivery systems [78,90]. Cubic and hexagonal phases provide a slow drug release matrix, and protect nucleic acids, proteins, and peptides from physical and chemical degradation, with a view to exploiting these features for small molecules such as acyclovir [109] and salbutamol [44]. For a given cubic phase, the rate of diffusion depends on the molecular size of the diffusant. Further, the rate of transport for a given diffusant can be adjusted when the aqueous channel size of the hosting cubic phase is changed by using lipids with different acyl chains. Additional control over the release was demonstrated when the strength of the electrostatic interaction between the diffusant and the walls of the channels were fine-tuned. Exquisite control over the release can be achieved by adjusting the electrostatic interaction’s strength and by his-tagged displacement [110].

## 9. Release, Absorption, and Permeation Mechanism

Drug colloidal carriers, especially LLC, seem to be promising candidates for the different administration routes and modified release of drugs, peptides, genes, and other bioactive compounds. Libster et al. first reported that the inverse hexagonal lyotropic mesophase could be used as a hydrophobic peptide crystallization medium, resulting in crystals of high specific quality which were stable for crystallographic analysis. In this study, cyclosporine-A crystals provided excellent X-ray information, with a 1.0 Å limit resolution diffraction [109].

A drug transdermal release system comprising the drug and a mesophase (amphipathic/lipophilic/water), or an expanded mesophase form, wherein the drug is contained within the mesophase, is a carrier adapted to transport and, after administration, gradually releases the drug in a sustained manner and for a long period of time [76,96].

Hexagonal mesophase drug release behavior seems to follow the Higuchi diffusion-controlled kinetic model [92], where the cumulative amount of drug diffusion through the matrix linearly depends on the square root of time. Geraghty et al. studied the in vitro antimuscarinic drug release from monoolein 18–99/water hydrogel, and found that sustained drug release for an 18-h period followed the Higuchi diffusion-controlled model [111]. Therefore, it seems that drug-controlled diffusion is influenced by the internal order and symmetry of mesophase.

Efrat et al. developed an analytical method to detect the levels in which LLC system host molecules contribute to mesophase’s internal order and symmetry (kosmotropic effect), and the level to which they destroy the internal symmetry and are phase transformation agents (chaotropic effect). In contrast, these authors reported that the presence of host molecule ions could be structurally complementary to one another and exhibit synergistic solubilization [70,98,112]. Apparently, the highest permeation rates can be obtained with molecules of higher ionic kosmotropic quality (OH^−^, CO_3_^2−^, SO_4_^2−^, HPO_4_^2−^, Mg^2+^, Li, Zn^2+^, and Al^3+^). In contrast, molecules with more chaotropic ions (Na^+^, K^+^, Br^−^, and I^−^) tend to be intercalated in the interfacial area and interact with the polar fraction of lipids. Thus, drug diffusion by the water channels of lyotropic mesophase is prevented by two factors [113]. The first is the drug geometric confinement, which restricts their movement and their diffusion in the LLC aqueous domain. The second factor is the chemical interaction’s strength between the drug’s and lipid’s hydrophilic and lipophilic fractions [114].

Using inverse hexagonal mesophase (H_II_), the authors have shown that small and hydrophilic peptides may be solubilized in the mesophase H_II._ In vitro studies demonstrated that hexagonal mesophase has the potential to release transdermal desmopressin and increase transdermal permeation of cyclosporine-A [100,101,115].

These systems have also been shown to be effective for target specific drug release. *p*-Aminobenzoic acid (PABA) is a drug used to treat skin fibrotic disorders and is also used as a sun screening agent. PABA has a limited oral bioavailability owing to its low solubility in water (6.1 mg·mL^−1^), which makes it a candidate drug for LC formulation. Kadhum et al. evaluated the utility of oral formulations to enhance PABA bioavailability and specific skin targeting. In this study, an oral formulation using the dispersions of LC cubosomes significantly increased LC-PABA bioavailability, when compared with PABA solution alone. The authors attributed the effect to LC-forming lipid types, suggesting that the oral administration of LC formulations is advantageous for the target specific release of PABA to the dermal tissue [116]. However, one must consider that the LC molecules are highly ordered, such as in solid state, whereas drug molecules in liquid preparations are free and diffuse randomly, reducing their bioavailability [117].

The rheological mucoadhesive properties of the LC precursor formulation for nasal administration comprising PPG-5-CE-TETH-20, oleic acid, and water, proved to be a promising strategy for the systemic release of zidovudine and other antiretroviral drugs [117]. The mucoadhesive LC precursor system comprising PPG-5-CETETH-20, oleic acid, chitosan, and poloxamer 407, was suitable for vaginal curcumin administration [106]. In both studies, drugs have their bioavailability limited by low water solubility. In the curcumin study, the LC form also increased the chemical stability of this phytochemical.

High concentrations of the polyethylene glycol oxy-stearate (HLB 14-16) and glycerol polyethylene glycol ricinoleate (HLB12-14) surfactants were used to prepare LLC nanoparticles to carry finasteride for topical delivery. Results showed dosage stability, a quick release, and a significant increase of finasteride dermal permeation. Permeation increase was attributed to the degree of lipid acyl chain disorganization, which might cause a fluidity increase in the stratum corneum.

The correlation between the structure of various LLC and anti-inflammatory diclofenac transdermal administration has been evaluated [89,92,118]. The Kosmotropic or chaotropic agents’ impact of diclofenac derived salts (Na^+^, K^+^, and diethylamine—DEA^+^) was assessed for different lyotropic mesophase structural characteristics and the transdermal release profile. The LC from this study was prepared by mixing monoolein/ethanol/water. The most chaotropic DEA effect between diclofenac derivatives (diclofenac DEA^+^) interacts with monoolein polar fraction and breaks its internal symmetry, causing phase transformation by expanding the lipid-water interface of lamellar and cubic mesophases. Potassium diclofenac, which is the least chaotropic diclofenac salt derivative, had a less pronounced effect on the mesophase structure. As expected, sodium salt (kosmotropic) had the least influence on both lamellar and cubic mesophases [115].

Another important application of LLC is the periodontal release of anesthetics and antibiotics for the prevention and treatment of infections. The interaction of lidocaine and lidocaine hydrochloride with the LC structure, for topical use, was studied. The in situ preparation of the anesthetic containing LC led to the arrest of the salt within the aqueous lamellar LC region, possibly owing to the chaotropic effect. In this case, the release of salt (lidocaine HCl) is slow, owing to the inability of the salt to penetrate more of the lipophilic fraction [119]. The various options described for the host drugs of microstructure LLC systems can be applied for analgesia, topical, ocular, post herpetic, and other applications [104,105,106].

The development of a pre-concentrate LLC-free water, with a good physical tolerance and the spontaneous formation of LC in an aqueous medium, is designed to be injected in the periodontal pocket, where it would be transformed into a crystalline phase of higher viscosity with a texture for controlled drug release. Metronidazole benzoate was used as an active agent. Miglyol 810 and polyethylenoglycol oxy-stearate (HLB 14–16), and glycerol polyethylenoglycol ricinoleate (HLB12-14), were used to form the LLC phase with 10%–40% water content. The rigidity of the lyotropic structure influenced by surfactants exerted a higher effect on the release of the metronidazole benzoate [119]. Bruschi et al. used a precursor system LC phase compound of gelatin microparticles, containing propolis, PPG-5-Ceteth 20, isopropyl myristate, and water for the treatment of periodontal diseases. In this study, the release was not Fickian (anomalous) for 10%, 30%, and 50% of the initial drug loading [120].

## 10. Conclusions

The alignment layers for LC and the creation of patterned surfaces by self-organization anisotropic molecules can be previewed in advance, using bottom-up approaches, for example. However, the complete control of adsorption on a surface is not easily achieved. As proposed by Hoogboom et al. one way to produce well-defined arrays is to use self-assembling molecules associated with physical phenomena, such as surface dewetting [121]. More detailed studies on this proposal could help overcome one of the main obstacles of clinical application and the business of the LLC forms.

The behavior of lyotropic biomimetic systems in drug delivery is multifactorial, such as the properties of the drugs, initial water content, phase LLC type, swellability, drug loading rate, the presence of ions with higher or less kosmotropic or chaotropic force, and the electrostatic interaction between the drug and the lipid bilayers. In this regard, it is a range that can be determined by the free energy of salt hydrogen bonds (ΔG_HB_). The higher the value of ΔG_HB_, the greater the kosmotropic effect, while the chaotropic effect is more pronounced for lower ΔG_HB_ values. Conversely, in vivo interaction studies between the LC and the drugs are limited, and the LC biopharmaceutical impact on the drugs’ bioavailability is not sufficiently clarified. The marketing of LLC systems still faces obstacles, including well defined methods for production scheduling, the loss of physical stability, and the absence of lipids with previously known phase behavior. However, scientific research on the mesophase systems continues to instigate researchers in both academia and industry. Studies not yet published by our group have shown an advance in knowledge to improve the clinical use of LLC in systems for drug delivery.
